# Photophysics and Cell Uptake of Self-Assembled Ru(II)Polypyridyl Vesicles

**DOI:** 10.3389/fchem.2020.00638

**Published:** 2020-07-30

**Authors:** Stephen Finn, Aisling Byrne, Karmel S. Gkika, Tia E. Keyes

**Affiliations:** School of Chemical Sciences and National Centre for Sensor Research, Dublin City University, Dublin, Ireland

**Keywords:** ruthenium(II)polypyridyl, vesicles, microscopy, cell-uptake, live cell fluorescence imaging, golgi apparatus, mitochondria

## Abstract

Effective delivery of luminescent probes for cell imaging requires both cell membrane permeation and directing to discrete target organelles. Combined, these requirements can present a significant challenge for metal complex luminophores, that have excellent properties as imaging probes but typically show poor membrane permeability. Here, we report on highly luminescent Ruthenium polypyridyl complexes based on the parent; [Ru(dpp)_2_(x-ATAP)](PF_6_)_2_ structure, where dpp is 4,7-diphenyl-1,10-phenanthroline and x-ATAP is 5-amino-1,10-phenanthroline with pendant alkyl-acetylthio chains of varying length; where x is 6; 5-Amido-1,10-phenanthroline-(6-acetylthio-hexanyl). 8; 5-Amido-1,10-phenanthroline-(8-acetylthio-octanyl). 11; 5-Amido-1,10-phenanthroline-(11-acetylthio-undecanyl); and 16; 5-Amido-1,10-phenanthroline-(16-acetylthio-hexadecanyl). Soluble in organic media, the alkyl-acetylthiolated complexes form nanoaggregates of low polydispersity in aqueous solution. From dynamic light scattering the nanoaggregate diameter was measured as 189 nm and 135 nm for 5 × 10^−6^ M aqueous solutions of [Ru(dpp)_2_(N∧N)](PF_6_)_2_ with the hexadecanoyl and hexanyl tails respectivly. The nanoaggregate exhibited dual exponential emission decays with kinetics that matched closely those of the [Ru(dpp)_2_(16-ATAP)]^2+^ incorporated into the membrane of a DPPC liposome. Cell permeability and distribution of [Ru(dpp)_2_(11-ATAP)]^2+^ or [Ru(dpp)_2_(16-ATAP)]^2+^ were evaluated in detail in live HeLa and CHO cell lines and it was found from aqueous media, that the nanoaggregate complexes spontaneously cross the membrane of mammalian cells. This process seems, on the basis of temperature dependent studies to be activated. Fluorescence imaging of live cells reveal that the complexes localize highly specifically within organelles and that organelle localization changes dramatically in switching the pendent alkyl chains from C16 to C11 as well as on cell line identity. Our data suggests that building metal complexes capable of self-assembling into nano-dimensional vesicles in this way may be a useful means of promoting cell membrane permeability and driving selective targeting that is facile and relatively low cost compared to use of biomolecular vectors.

## Introduction

Ruthenium(II) polypyridyl complexes are attractive cell imaging probes and potential phototherapeutics because of their amenable optical and redox properties coupled with their synthetic versatility. Compared to organic fluorophores, more conventionally used in cell imaging, Ruthenium(II) polypyridyl complexes exhibit exceptionally long-lived emission from their triplet (dπ-π^*^) MLCT states that can facilitate environmental sensitivity toward for example pH and O_2._ Such parameters are valuable indicators of the metabolic status of the living cell (Carlsson et al., 2000; Ji et al., 2002; Zhong et al., 2003; Margineanu et al., 2007; Neugebauer et al., 2008). Ruthenium(II) polypyridyl complexes also typically exhibit good photostability rendering them suitable for repeat or long-term dynamic imaging experiments (Byrne et al., 2016) Their large Stokes shifts mean that even at high concentrations, for example under conditions of high localization, they are free from artifactual effects that can affect fluorescence, such as inner filter or self-quenching effects (Bailey and Cullis, 1997; Margineanu et al., 2007). Ruthenium polypyridyl complexes typically emit in the red to NIR spectral region, well resolved from any autofluorescence in biological media.

From a therapeutic perspective, ruthenium polypyridyl complexes have been widely studied with nucleic acid materials, and a range of complexes have demonstrated ability to bind and cleave DNA under irradiation, through both type I and type II mechanisms (Zeglis et al., 2007; Brabec and Kasparkova, 2018; Saeed et al., 2020).

However, a key limitation to the application of such complexes in bioimaging is their limited ability to cross the cell membrane without the requirement for permeabilization. Membrane permeabilization can be readily achieved through applying the luminophore to the cell along with organic solvent such as dimethyl sulfoxide (DMSO) or ethanol, or through the use of detergent, but such approaches are not ideal as they disrupt the plasma membrane, and have no capacity for directing the complex within the cell. Furthermore, such methods are not typically suitable for tissue or *in-vivo* imaging applications. A number of approaches have been demonstrated to achieve reliable permeation and indeed specific organelle targeting of complexes within the living cell. These include modifying compound charge and hydrophilicity or conjugation of biological moieties to the probe (Zhang and Lo, 2009; Pisani et al., 2010; Li et al., 2013; Lo, 2015; Zabarska et al., 2016; Caporale et al., 2017; Chakrabortty et al., 2017; Dolan et al., 2017; Caporale and Massi, 2018). Our work has focused on the use of bioconjugation, especially of cell penetrating (CPP) and signal peptides and we have shown, that this can be a highly effective means of targeting (Dolan et al., 2017; Hahn et al., 2017) However, the associated synthesis and purification is expensive and time consuming.

Nanocarriers are a widely studied tool in drug delivery, and an attractive proposition both for transport of ruthenium probes across the cell membrane and for achieving targeting. Indeed, one may amplify the intensity of the luminophore through incorporation of ruthenium polypyridyl complexes into amphiphilic nanovectors (Ellahioui et al., 2019; Liang et al., 2020; Saeed et al., 2020).

Where amphiphilic Ru complexes are able to form self-assembled vesicles, the probe concentration may be maximized, avoiding self-quenching because of their large Stokes shift, to deliver high brightness nanoparticles capable of penetrating the cell membrane. The impact of association with micelles and vesicles on the photophysics of Ru(II) polypyridyl has been widely explored (Draeger et al., 2000; Bowers et al., 2003; Jebb et al., 2007; Guerrero-Martínez et al., 2008; Barbante et al., 2011; Hansen et al., 2014; Limburg et al., 2016). Whereas metal complex luminophore association (Hansen et al., 2014) with liposomes in the biological context has only relatively recently been investigated (Gaines, 1980; Gutiérrez et al., 2003; Mechler et al., 2014; Patra et al., 2016). For example, Shen et al. (2017) demonstrated the application of a liposome loaded with Ruthenium (II) polypyridyl in promoting uptake of the complex into cancer cells and accumulation in tumor *in-vivo*. Formation of supramolecular aggregates by amphiphilic metal luminophore, as described, is interesting because of the high concentration of luminophore achievable and has been described for a number of ruthenium and iridium complexes, especially from the perspective of their optical applications in photovoltaics (Guerrero-Martínez et al., 2008; Nehru et al., 2017). However, the application and implications of such materials in bioimaging has not been as widely studied to date.

Reports on Ir(III) luminophores have shown that metal complex aggregates can be cell permeable. For example, dendritic structures have been described for Ir(III) complexes (Zhang et al., 2010) and shown to fuse with cell membranes. Such fusion is thought to be an important step in the mechanism of viral penetration of mammalian cells (Bailey and Cullis, 1997; Kanaseki et al., 1997). Lo et al. demonstrated a series of iridium polypyridyl complexes with pendent alkyl chains that associated with liposomes of DSPC (1,2-distearoyl-*sn*-glycero-3-phosphocholine) (Lo et al., 2008). These complexes were shown to be taken up into the cytoplasm of live HeLa cells after a 5-h incubation. Coogan *et al* also reported on a series of rhenium complexes with pendent alkyl chains (Amoroso et al., 2007). On incubation of *Spironucleus vortens* cells with the complexes for 2 h, fluorescence intensity imaging confirmed the presence of the complexes intracellularly.

Herein, we report the synthesis and characterization of a family of dpp (4,7-diphenyl-1,10-phenanthroline) containing complexes with pendant alkyl-acetylthio chains of varying length; [Ru(dpp)_2_(x-ATAP)](PF_6_)_2_, where x-ATAP is 5-amino-1,10-phenanthroline; where x is 6; 5-Amido-1,10-phenanthroline-(6-acetylthio-hexanyl). 8; 5-Amido-1,10-phenanthroline-(8-acetylthio-octanyl). 11; 5-Amido-1,10-phenanthroline-(11-acetylthio-undecanyl); and 16; 5-Amido-1,10-phenanthroline-(16-acetylthio-hexadecanyl).

It was predicted that complexes, with acetylthio-alkyl tails might act as metallosurfactants leading to aggregation in aqueous media that might promote permeation of the lipid bilayer of the cell in a similar manner to a liposome, possibly facilitating the uptake of [Ru(dpp)_2_(x-ATAP)](PF_6_)_2_ into the cytoplasm. We evaluate herein the photophysics the [Ru(dpp)_2_(x-ATAP)](PF_6_)_2_ family in water and organic solvents and observe, that whilst forming a homogenous solution in the latter, they form nanoaggregates in the former whose size from dynamic light scattering, varies with chain length. Selecting the C11 and C16, the properties of the aggregates are studied by steady state and time resolved luminescence, a critical micelle concentration is estimated and the interaction of aqueous solutions of the nanoaggregates with live cells are evaluated. With live cells we observe rapid, activated uptake at physiological temperature that is suppressed at 4°C. While both aggregates are readily membrane permeable, we observed some intriguing differences in localization with chain length.

## Materials and Methods

### Materials

All reagents used in synthesis were analytical grade. Absorption and emission spectroscopy were carried out in spectroscopic grade acetonitrile or dichloromethane or purified water. Solution phase electrochemistry was carried out in spectroscopic grade acetonitrile. Water was purified using a MilliQplus−185 Millipore system. Chemicals were purchased from Sigma-Aldrich and were used as received.

### Instrumentation

^1^H NMR was recorded on a Bruker Avance Ultrashield 400 spectrometer using the solvent-proton as an internal standard. Electrospray (ESI)-mass spectrometry (MS) data were recorded on a Brüker Esquire 400 LC–MS, by direct injection on electrospray positive mode. Elemental analyses were performed by UCD microanalysis laboratory.

Absorption spectra were recorded using a Varian Cary 50 spectrometer. Steady state emission spectra were measured on a Varian Cary Eclipse spectrometer. Luminescent lifetimes were recorded on a Picoquant Nanoharp time correlated single photon counting spectrometer. Particle sizing was carried out by dynamic light scattering on a Delsa Nano C Submicron Particle Size and Zeta Potential Particle Analyzer with the standard size cell accessory. Confocal imaging was carried out using a Leica TSP DMi8 confocal microscope with a 100X oil immersion objective lens.

Electrochemistry was carried out on a CH Instruments 660 potentiostat using a three-electrode cell comprising a 3 mm diameter glassy carbon electrode working electrode, platinum wire counter electrode and Ag/AgNO_3_ non-aqueous reference electrode. Solution phase electrochemistry was conducted in an electrolyte of 0.1 mM TBA ClO_4_ in acetonitrile. The reference electrode was calibrated using the Fc+/Fc ferrocene redox couple at +0.64 V and all potentials are quoted vs. Ag/AgNO_3_ electrode.

### [Ru(dpp)_2_(16-ATAP)]^2+^ Modified DPPC Liposome Preparation

One mL of a 100:1 mix of DPPC (dipalmitoylphosphatidylcholine) phospholipid and Ru(dpp)_2_(16-ATAP)]^2+^ (1 μM) were combined in chloroform in a glass vial. The solvent was stripped with a flow of N_2_ leaving a thin layer of lipid on the walls of the glass vial. The lipid film was resuspended in 1 mL of phosphate buffer (pH 7.4) and sonicated for 10 min to ensure the formation of unilamellar vesicles. The liposome solution was then extruded through a 100 nm polycarbonate filter 5 times at 60°C using an Avanti Mini-Extruder system to obtain uniform dimensioned liposomes.

### Cell Culture

HeLa cells, a cervical cancer cell line, were cultured in MEME media supplemented with 10% fetal bovine serum, 2% L-glutamine, 1% MEM non-essential amino acid solution, and 1% penicillin-streptomycin and grown at 37°C with 5% CO_2_. Chinese hamster ovary (CHO) mammalian cells were cultured in DMEM/F-12 Hams 50/50 mix, supplemented with 10% fetal bovine serum and 1% penicillin-streptomycin and grown at 37°C with 5% CO_2_. Cells were harvested or split at 90% confluency using 0.25% trypsin for 5 min at 37°C. All studies were repeated *n* = 3.

### Confocal Imaging

HeLa cells were seeded at 7.5 × 10^4^ cells in 2 mL culture media on 35 mm high precision glass-bottom dishes (Ibidi, Germany) and left for 48 h at 37°C under 5% CO_2_. [Ru(dpp)_2_(16-ATAP)]^2+^ and [Ru(dpp)_2_(11-ATAP)]^2+^ were added to the wells in cell media to give a final concentration of 2.5 μM (final DMSO concentration of 0.5%) and were incubated for 24 h at 37°C with 5% CO_2_. Prior to imaging, the compounds were removed, and the cells were washed once with PBS supplemented with 1.1 mM MgCl_2_ and 0.9 mM CaCl_2_. The cells were imaged live using a Leica TSP DMi8 confocal microscope with a 100X oil immersion objective lens. A heated box covered the stage to maintain the temperature at 37°C. A 470 nm laser was used to excite the compounds, and the emission was collected between 520 and 620 nm. For co-localization studies, cells stained with the compounds were incubated with MitoTracker Deep Red (Thermo Fisher) (75 nM) for 20 min at 37°C, washed, and imaged. MitoTracker Deep Red was excited at 633 nm and emission was collected between 645 and 745 nm. For co-localization studies in the Golgi apparatus, Cell Light™ Golgi-RFP BacMam 2.0 (Thermo Fisher) was added to live cells at 30 μL in 2 mL cell media for 24 h. The dye/media was removed, and cells were washed twice with PBS and imaged. Golgi-RFP was excited at 555 nm and emission was collected at 584 nm.

To assess the mode of uptake, cells were prepared as described above and incubated with the compounds at 4°C for 4 h. Cells were washed with PBS (supplemented with 1.1 mM MgCl_2_ and 0.9 mM CaCl_2_) and imaged immediately.

## Results and Discussion

### Synthesis

Detailed synthesis and structural characterization of the heteroligands, x-ATAP, and the complexes [Ru(dpp)_2_(x-ATAP)](PF_6_)_2_ are provided in Supplemental Materials. The x-ATAP ligand was prepared from hydrazine reduction of 5-nitro-1,10 phenanthroline to yield the amino precursor. This was peptide coupled to 4- 8-(acetylthio)-X acid derivatives; where x is hexanonic, octanonic undecanoic and hexadecenoic acid, via DMTMM (4-(4,6-Dimethoxy-1,3,5-triazin-2-yl)-4-methyl-morpholinium) coupling. The four [Ru(dpp)_2_(x-ATAP)](PF_6_)_2_ complexes were then readily prepared through reflux of the dichloride precursor in ethanol/water Ru(dpp)2Cl2, with each x-ATAP ligand according to previously reported methods (Blackmore et al., 2013; Adamson et al., 2014). The complexes were characterized by ^1^HNMR and by elemental analysis and conformed to expected values.

### Photophysics

**Figure 1:**
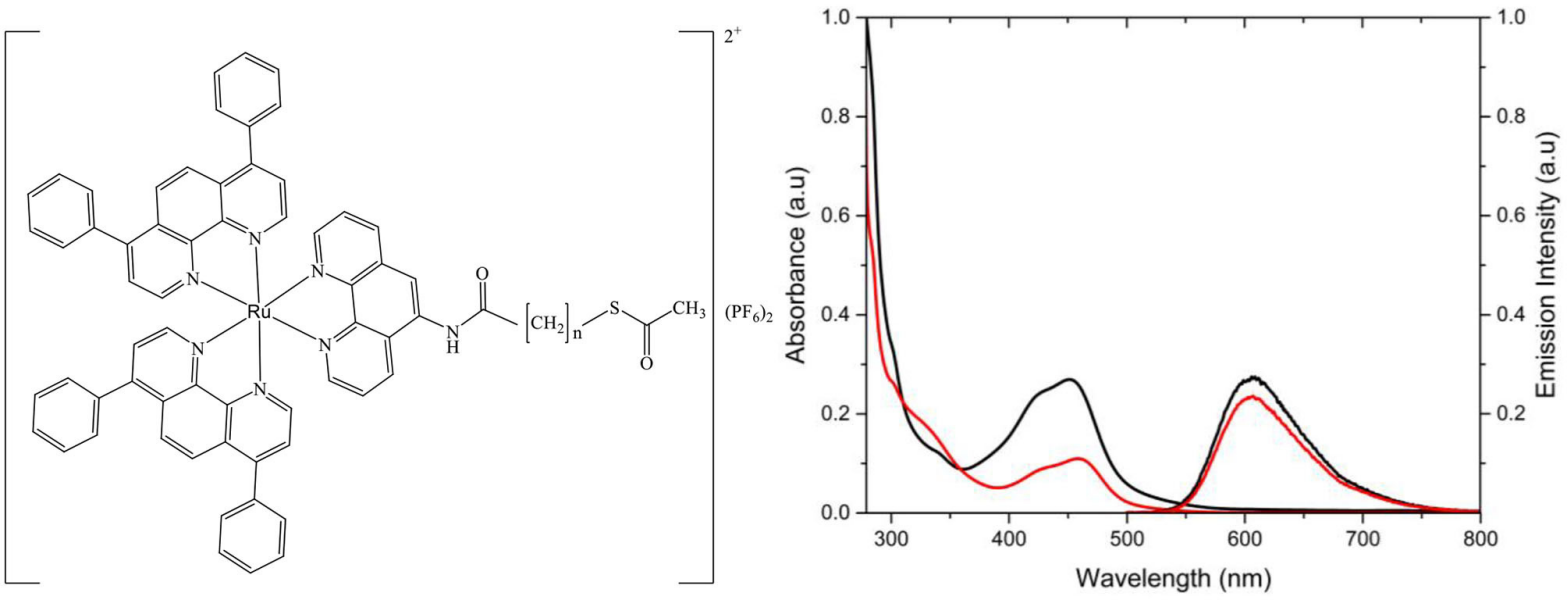
Structure of [Ru(dpp)_2_(16-ATAP)](PF_6_)_2_ where x is the number of carbons in the aliphatic chain, resulting in [Ru(dpp)_2_(16-ATAP)]^2+^ or [Ru(dpp)_2_(11-ATAP)]^2+^. Absorbance and emission spectra of [Ru(dpp)_2_(11-ATAP)]^2+^ (^**__**^) and [Ru(dpp)_2_(16-ATAP)]^2+^ (^**__**^) in PBS (15 μM, λ_exc_ 454 nm; slit width = 5 nm).

The optical and photophysical properties of the complexes in acetonitrile are presented in Table 1. The spectroscopy is as reported previously for Ruthenium(II) polypyridyl complexes, the UV spectral region is dominated by ligand based optical transitions and a d-d transition around 315 nm (Juris et al., 1988). The characteristic ^1^MLCT Ru (II) → (ligand) absorption is centered at approximatley 454 nm (Crosby et al., 1965; Bachas et al., 1997; Kim et al., 1997; Mongey et al., 1997). And, as expected, the visible electronic absorption for each complex is identical within experimental error, representative spectra for chain length C16 and C11 are shown in Figure 1. Resonance Raman spectroscopy exciting at 457.8 nm is idenitcal in each case also confirming the origin of the absorbance is unaffected by the thioacetate appendage (Figure S5, Supplemental Materials). The complexes exhibit intense emission centered at λ_em_ = 608 nm from the ^3^MLCT. Emission spectra for the complexes under absorbance matched excitation are shown and indicate that the quantum yield is the same in acetonitrile, at 0.0114 ± 0.0002, irrespective of alkyl chain length, within experimental error. The Stokes shift, the energy gap between the lowest energy absorption and the emission maximum, was calculated to be 154 nm which is slightly greater than that of [Ru(bpy)_3_]^2+^.

**Table 1:** Photophysical properties of the [Ru(dpp)_2_(x-ATAP)](PF_6_)_2_ complexes in various solvents in aerated conditions at 298 K.

	**Absorbance (nm)**		**Emission (nm)**				**Emission**		**Lifetime**	
	**MeCN**	**DCM**	**H_**2**_O**	**MeCN (Φ)**	**DCM**	**H_**2**_O**	**MeCN**	**DCM**	**H**_****2****_**O (biexponential)**	
[Ru[(dpp)_2_(NH_2_phen)]^2+^	454	460	–	617 (0.0094 ± 0.0004)			1.62E-07 ± 1.7E-08			
[Ru(dpp)_2_(6-ATAP)]^2+^	454	459	438	608 0.0114 ± 0.0002	596	628	1.75E-07 ± 2.2E-08	6.46E-07 ± 3.8 E-09	7.38E-07 ± 2.3E-09 (91 %)	1.05E-07 ± 1E-09 (9 %)
[Ru(dpp)_2_(8-ATAP)]^2+^	454	459	438	608 0.0114 ± 0.0002	596	636	1.73E-07 ± 1E-08	6.39E-07 ± 8E-09	8.29E-07 ± 2.5E-10 (90 %)	1.35E-07 ± 2.5E-10 (10 %)
[Ru(dpp)_2_(11-ATAP)]^2+^	454	459	438	608 0.0114 ± 0.0002	596	636	1.82E-07 ± 2.3E-08	6.42E-07 ± 4.8E-09	8.06E-07 ± 1E-09 (89 %)	1.37E-07 ± 1.8E-09 (11 %)
[Ru(dpp)_2_(16-ATAP)]^2+^	454	459	438	608 0.0114 ± 0.0003	596	618	1.85E-07 ± 2.6E-08	6.39E-07 ± 1.28E-08	9.19E-07 ± 7.5E-09 (90 %)	1.38E-07 ± 4E-09 (10 %)

*In all cases the concentration of [Ru(dpp)_2_(x-ATAP)](PF_6_)_2_ was 5 μM*.

The emission intensity and lifetime of these complexes is very oxygen sensitive. As shown in Table 1, in acetonitrile, the emission of all [Ru(dpp)_2_(x-ATAP)](PF_6_)_2_ decays according to single exponential kinetics with τ of ~175 ns under aerated conditions across all alkyl chain lenghts increasing by approximately an order of magnitude to ~1 μs in dearated acetonitrile, comparable oxygen sensitivity to quantum yields are also observed.

The alkyl chain length, excerts no impact on the optical properties or photophysics in non-aqueous media, k_r_ and k_nr_ values are the same as across the [Ru(dpp)_2_(x-ATAP)]^2+^ series, within experimental error. The high sensitivity of luminescence lifetime and quantum yield of [Ru(dpp)_2_(x-ATAP)](PF_6_)_2_ complexes to [O_2_] observed in organic media is consistent with previous reports on photophysical properties of ruthenium complexes containing dpp ligands (Crosby and Watts, 1971; Demas et al., 1977; Carraway et al., 1991; Draxler et al., 1995). This sensitivity makes the [Ru(dpp)_2_(x-ATAP)]^2+^ family potentially good candidates as O_2_ probes for O_2_ concentration mapping in complex matrices such as cells. However, as described below, behavior in aqueous solution is complicated due to aggregation of the complexes.

### Electrochemistry

Solution phase electrochemistry was carried out on 1 mM solutions of complex in ACN with 0.1 mM TBATBF_4_ as the supporting electrolyte at a glassy carbon working electrode. Table S1, Supplemental Materials, summarizes the solution phase electrochemical properties of the four [Ru(dpp)_2_(x-ATAP)]^2+^ complexes. The voltammetry of the complexes is very similar and a representative cyclic voltammogram for the [Ru(dpp)_2_(16-ATAP)](PF_6_)_2_ is shown in Figure S5. A reversible one electron oxidation with an E_1/2_ value of +0.92 V is attributed to the Ru^2+/3+^ couple. Ligand based reductions are observed between −1.6 and −2 V, and are somewhat poorly resolved but integrate for 3, 1 electron processes. The potential of the Ru^2+/3+^ redox couple of [Ru(dpp)_2_(ATAP)]^2+^ at +0.92 V are more positive than reported for [Ru(bpy)_3_]^2+^. This is as expected on the basis of the increased π-acceptor properties of the dpp ligand and is also reflected in the more positive first ligand reduction at −1.7 V compared to the ligand reduction at −1.76 V of the first ligand reduction of [Ru(bpy)_3_]^2+^.

Notably, an intense, and irreversible adsorption peak is observed in the CVs of all [Ru(dpp)_2_(x-ATAP)]^2+^ complexes at approximately −2.1V. Based on its characteristic sharpness, for the parent [Ru(dpp)_2_(ATAP)]^2+^ complex such a peaks is not obesrved and is attributed to deposition of [Ru(dpp)_2_(x-ATAP)]^2+^ at the carbon electrode surface. Adsorption may be due to the surfactant-like behavior of the complex or may be via thiol grafting to glassy carbon, as has been noted previously for thiols and in the present case, maybe mediated through reduction of the thioacetate to thiol (Médard and Morin, 2009; Pchelintsev et al., 2011).

### Solvent Dependence and Aggregate Formation

**Figure 2:**
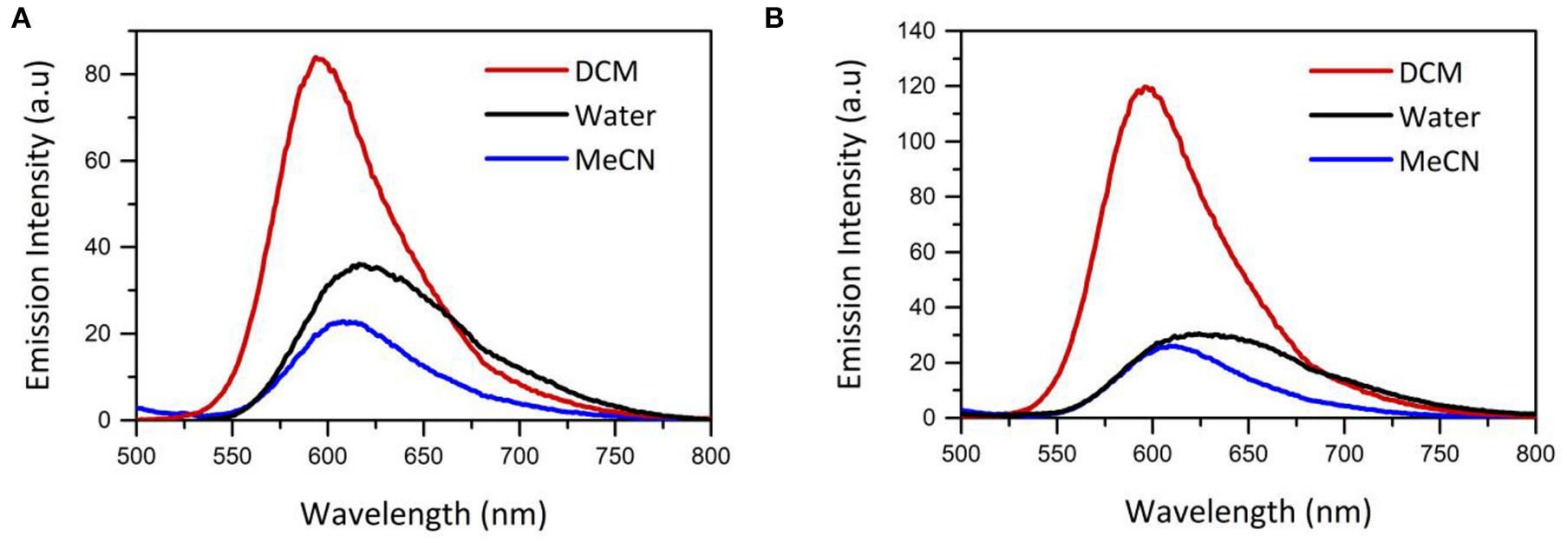
Impact of solvent on emission spectrum of **(A)** [Ru(dpp)_2_(16-ATAP)]^2+^ **(B)** [Ru(dpp)_2_(6-ATAP)]^2+^ shown in MeCN, DCM and H_2_O. Concentration of Ru(dpp)_2_(16-ATAP)]^2+^ is 5 × 10^−6^ M in all solvents. Excitation wavelenght = 454 nm. *n* = 3.

The photophysical properties of the [Ru(dpp)_2_(x-ATAP)]^2+^ series were compared in aerated MeCN, DCM and H_2_O, and data is presented in Table 1, and the emission spectra of the [Ru(dpp)_2_(x-ATAP)]^2+^ complexes in DCM, acetonitrile and water are compared in Figure 2.

As the neat complexes were poorly soluble in aqueous solution, they were first dissolved in minimum acetonitrile and then made up in aqueous solution to yield a homogeneous solution in 99/1 % V/V water/acetonitrile.

The λ_max_ of the MLCT absorption band of the [Ru(dpp)_2_(x-ATAP)]^2+^ complexes is minimally affected by solvent in DCM compared to acetonitrile. In water, the absorption band is slightly red shifted but broadened with some shift in baseline which can be attributed to the formation of aggregates. Emission is more sensitive to solvent effects. The λ_max_ of emission for the [Ru(dpp)_2_(x-ATAP)]^2+^ complexes in DCM is centered at 596 nm and red shifts to 608 nm in acetonitrile, but the quantum yield of emission is dramatically affected by solvent, increasing by approximately a factor of 5 in DCM compared to acetonitrile and correspondingly, the emission lifetime, which fits a single exponential decay in organic media, increases from an average of 179 ns in ACN to 642 ns in dichloromethane (aerated media). Within experimetal error, these values did not vary with alkyl chain length. Similar solvatochromic effects have been reported for related ruthenium polypyridyl complexes by Sun and Turro (2010). This was attributed to assignment of lowest energy excited state to a triplet MLCT where the charge is localized on dppp ligands rendering the complexes sensitive to solvent polarity.

Conversely the emission spectrum of [Ru(dpp)_2_(x-ATAP)]^2+^ changes dramatically in water, where it broadens and is red shifted λ_max_ compared to DCM and acetonitrile. Interestingly, the extent of red shift varies with chain length, the first example of where impact of chain length impacts optical properties for the complexes, where the red shift is much less pronounced for [Ru(dpp)_2_(16-ATAP)]^2+^, at 618 nm compared to other chain lengths where peak intensity is observed around 640 nm.

Notably, in water the emission decay profile changes from a single to bi-exponential decay, where the long lifetime component which constitutes ~90% of the decay amplitude is ~800 ns and the short lifetime component of about 130 ns constitutes the remaining 10% of amplitude. The short lifetime is attributed to solvated [Ru(dpp)_2_(x-ATAP)]^2+^, as it is similar to lifetimes of related dpp coordination compounds of ruthenium in water under aerated conditions (Blackmore et al., 2013). The long lifetime component is unexpected on the basis of the known behavior of dpp based complexes in aerated aqueous media, but is comparable, along with the dual exponential profile of the decay to behavior reported in Ru(II) dpp complexes when bound in non-aqueous structures within water such as DNA or lipid bilayers (Barton et al., 1986; Adamson et al., 2014). Given the relative hydrophobicity of these complexes and their long alkyl chains, the anomalous photophysical behavior, we ascribe the behavior to formation of luminescent aggregates in aqueous solution, *vide infra*. To evaluate the impact of aggregate formation on the photophysical properties of the complex we prepared 100 nm DPPC liposomes into which the [Ru(dpp)_2_(x-ATAP)]^2+^ C16 complex was embedded, by preparing the liposomes from lipid mixed with complex, the lipophillic tail is expected to confine it to the lipid membrane. Strongly consistent with data for [Ru(dpp)_2_(x-ATAP)]^2+^ in aqueous media, the emission decay from the [Ru(dpp)_2_(16-ATAP)]^2+^ within liposomes conformed to biexponential kinetics. The main component, τ_1_ exhibted a decay of 1.18 μs with τ_2_ of 201 ns (Supplemental Materials, Figure S6).

Such multi-exponential lifetimes of ruthenium complexes in aggregate environments have been noted for vesicular Ruthenium complexes. De Cola et al. reported the biexponential lifetimes of dialkyl ruthenium complexes that form micelles which have a long and short emission lifetime component, attributed to micellular and solvated ruthenium complex respectively (Guerrero-Martínez et al., 2008). Multi-vesicular structures of ruthenium complexes with long alkyl chains were also reported by Fuhrhop et al. and shown to exhibit tri-exponential lifetimes (Draeger et al., 2000). Overall, comparison between liposome encapsulated and complexes in water indicates strongly that the complexes are self-assembling into micellar or bicellar aggregates.

**Figure 3:**
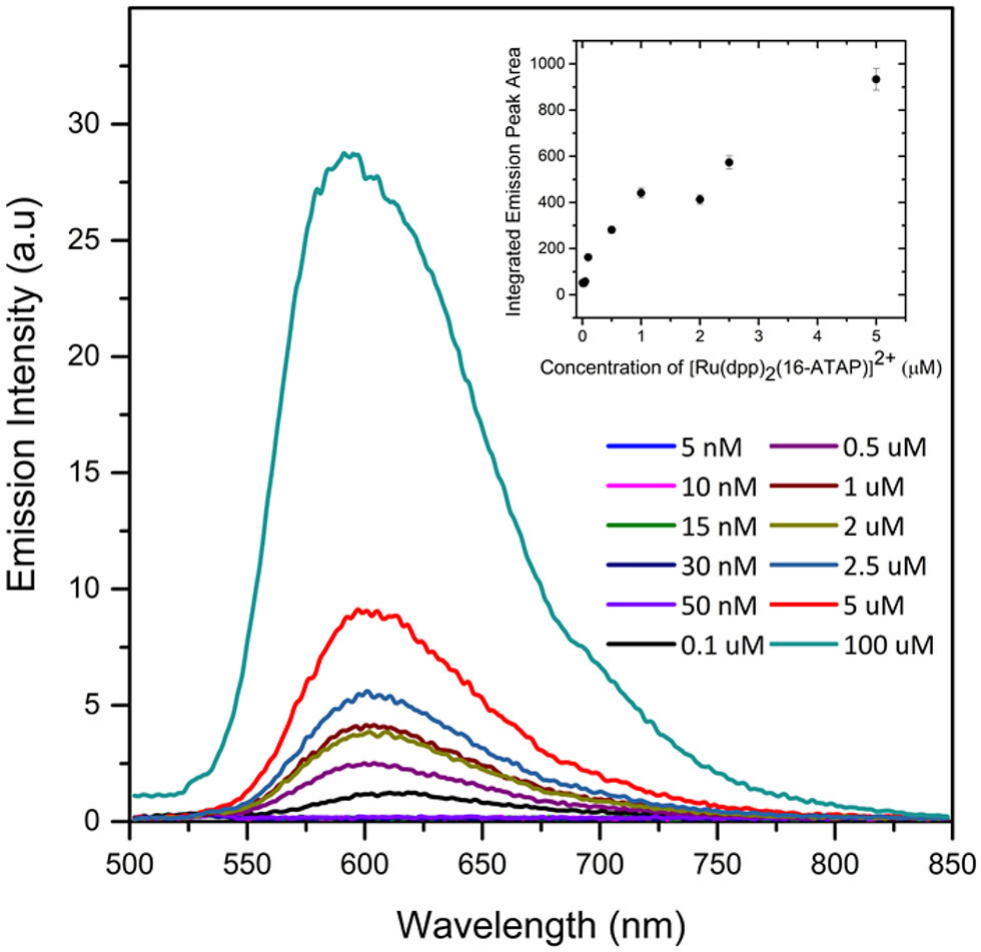
Concentration dependence of emission spectroscopy of [Ru(dpp)_2_(16-ATAP)]^2+^ in aqueous PBS buffer (1% v/v ACN) and plot of integrated area of [Ru(dpp)_2_(16-ATAP)]^2+^ as a function of concentration (inset) (λ_exc_ 454 nm; excitation and emission slit width 5 nm; *n* = 3).

We evaluated the concentration dependence of the emission intensity for [Ru(dpp)_2_(16-ATAP)]^2+^ in aqueous solution to further confirm aggregate formation, and to make an estimate of the critical micelle concentration (CMC). Figure 3 shows the experimental result and the inset plots the integrated emission area vs. concentration. This approach was taken as, notably the emission λ_max_ blue shifts from 612 nm at 0.1 μM to ~600 nm above this concentration. And correspondingly, this concentration corresponds to the point where the emission intensity deviates from linearity, and is taken to be approximately the CMC.

To evaluate the size and polydispersity of the resulting nanoaggregates, we studied aqueous solutions of [Ru(dpp)_2_(x-ATAP)]^2+^ by dynamic light scattering (DLS). The DLS data are tabulated in Table 2. For aqueous 5 × 10^−6^ M solutions of complex [Ru(dpp)_2_(11-ATAP)]^2+^ and Ru(dpp)_2_(16-ATAP)]^2+^ in H_2_O. The solutions gave a strong scattering signal consistent with the formation of particle aggregates, while in contrast, solutions of [Ru(dpp)_2_(11-ATAP)]^2+^ and Ru(dpp)_2_(16-ATAP)]^2+^ in acetonitrile and dichloromethane gave no scattering signal indicating as expected that the complexes dissolved to give homogenous solutions in these solvents.

**Table 2:** DLS and Zeta potential measurements of [Ru(dpp)_2_(16-ATAP)]^2+^ (5 μM) and [Ru(dpp)_2_(16-ATAP)]^2+^ (5 μM) in H_2_O and PBS buffer (pH 7.4).

**Sample**	**Size (nm)**	**PDI**
[Ru(dpp)_2_(16-ATAP)]^2+^ (PBS)	189.92 ± 2.13	0.328 ± 0.045
[Ru(dpp)_2_(16-ATAP)]^2+^ (H_2_O)	240.90 ± 5.07	0.558 ± 0.052
[Ru(dpp)_2_(11-ATAP)]^2+^ (PBS)	146.67 ± 7.21	0.525 ± 0.089
[Ru(dpp)_2_(16-ATAP)]^2+^ (H_2_O)	163.80 ± 4.56	0.502 ± 0.037
[Ru(dpp)_2_(6-ATAP)]^2+^ (H_2_O)	135.3 ± 0.8	0.122 ± 0.023

*Measurements were performed in triplicate (n = 3)*.

DLS measurements showed that the diameter of the aggregates ranged from 249 to 135 nm for 5 × 10^−6^ M solutions of the complexes in water and aqueous PBS buffer. The polydispersity indices were between 0.1 and 0.5 indicating good monodispersity in size distribution of the aggregates. The large hydrodynamic radii of the aggregates suggest that they are likely bilayer or multivesicular rather than micellear strucutres. The zeta potential was measured for the [Ru(dpp)_2_(16-ATAP)]^2+^ and [Ru(dpp)_2_(11-ATAP)]^2+^ respectively as these were the structures that we focused on for imaging studies.

### Cell Uptake Studies

Given the complexes form vesicles in aqueous media we were interested to investigate if these aggregates are cell permeable, and thus if their assembly into vesicles in aqueous solution promoted uptake and localization in live cells. For cell studies we focused on [Ru(dpp)_2_(16-ATAP)]^2+^ and [Ru(dpp)_2_(11-ATAP)]^2+^ and evaluated their uptake in live HeLa and CHO cells by incubating the complexes with the cells at final complex concentrations each of 2.5 μM in cell growth media for 24 h in the absence of light. This concentration was used as it is well in excess of the value estimated for CMC ensuring the complexes are primarily present as aggregates. After incubation with the complex, the cells were washed twice with PBS (supplemented with 1.1 mM CaCl_2_ and 0.9 mM MgCl_2_) and imaged using confocal laser scanning microscopy (CLSM).

**Figure 4:**
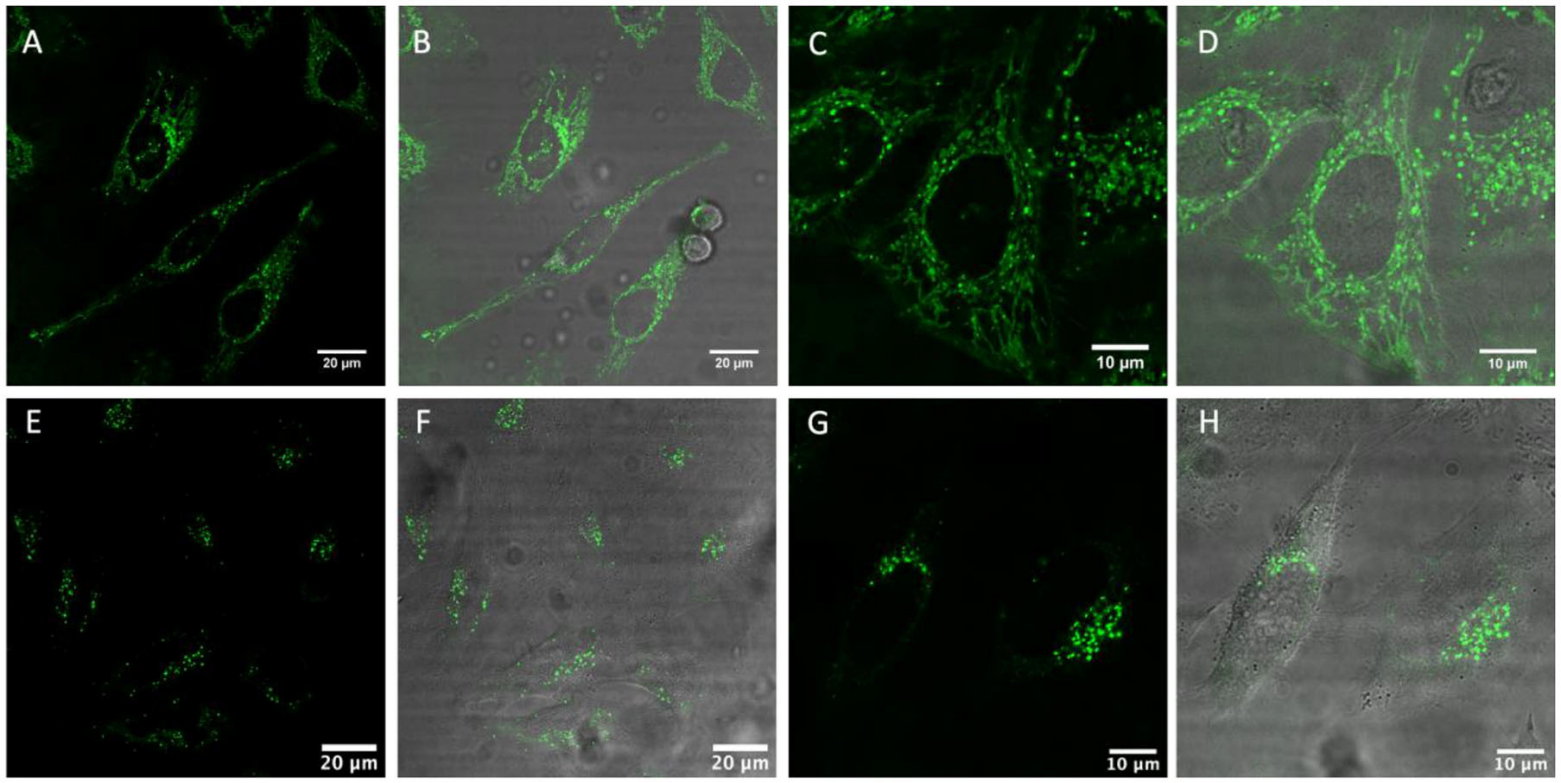
Live HeLa cells stained with [Ru(dpp)_2_(16-ATAP)]^2+^ **(A–D)** and [Ru(dpp)_2_(11-ATAP)]^2+^ **(E–H)** at 2.5 μM for 24 h. **(A,C,D,E)** show the ruthenium emission channel, and **(B,D,F,H)** shows the overlay of the ruthenium channel with brightfield channel.

Figure 4 shows live HeLa cells post incubation with [Ru(dpp)_2_(11-ATAP)]^2+^ and [Ru(dpp)_2_(16-ATAP)]^2+^. Both complexes emit intensely from within the cells with no background contribution. Both complexes are nuclear excluded and appear to localize very discretely but at distinctive regions of the cell. Figures 3A–D shows [Ru(dpp)_2_(16-ATAP)]^2+^ accumulates in the cytoplasm, and localizes in what appears to be the mitochondria. While interestingly, [Ru(dpp)_2_(11-ATAP)]^2+^ has a completely different distribution, emitting as a pattern of punctate spherical objects highly localized to one side of the nucleus (E–F). Such a pattern is suggestive of the Golgi Apparatus labeling. It is important to note that when incubated at 4°C, neither [Ru(dpp)_2_(16-ATAP)]^2+^ nor [Ru(dpp)_2_(11-ATAP)]^2+^ entered the cells (ESI Figures S9, S10), indicating that the uptake is via an energy-dependent mechanism such as endocytosis.

This conclusion is further supported by the punctate appearance of the cytoplasmic labeling, this is characteristic of endocytic uptake (Puckett et al., 2010), which was observed throughout the uptake studies.

Remarkably, when incubated with CHO cells, under identical conditions, while both complexes show similar cell permeability, the [Ru(dpp)_2_(16-ATAP)]^2+^ and [Ru(dpp)_2_(11-ATAP)]^2+^ distributed in a completely different manner to HeLa cells (ESI Figure S11). In CHO cells, both complexes distribute throughout the cytoplasm with punctate features that appear to be endosomes associated with endocytosis, Fluorescence lifetime imaging also confirms this distribution (shown in Figure S14). This pattern remains unchanged over time with no further localization evident.

**Figure 5:**
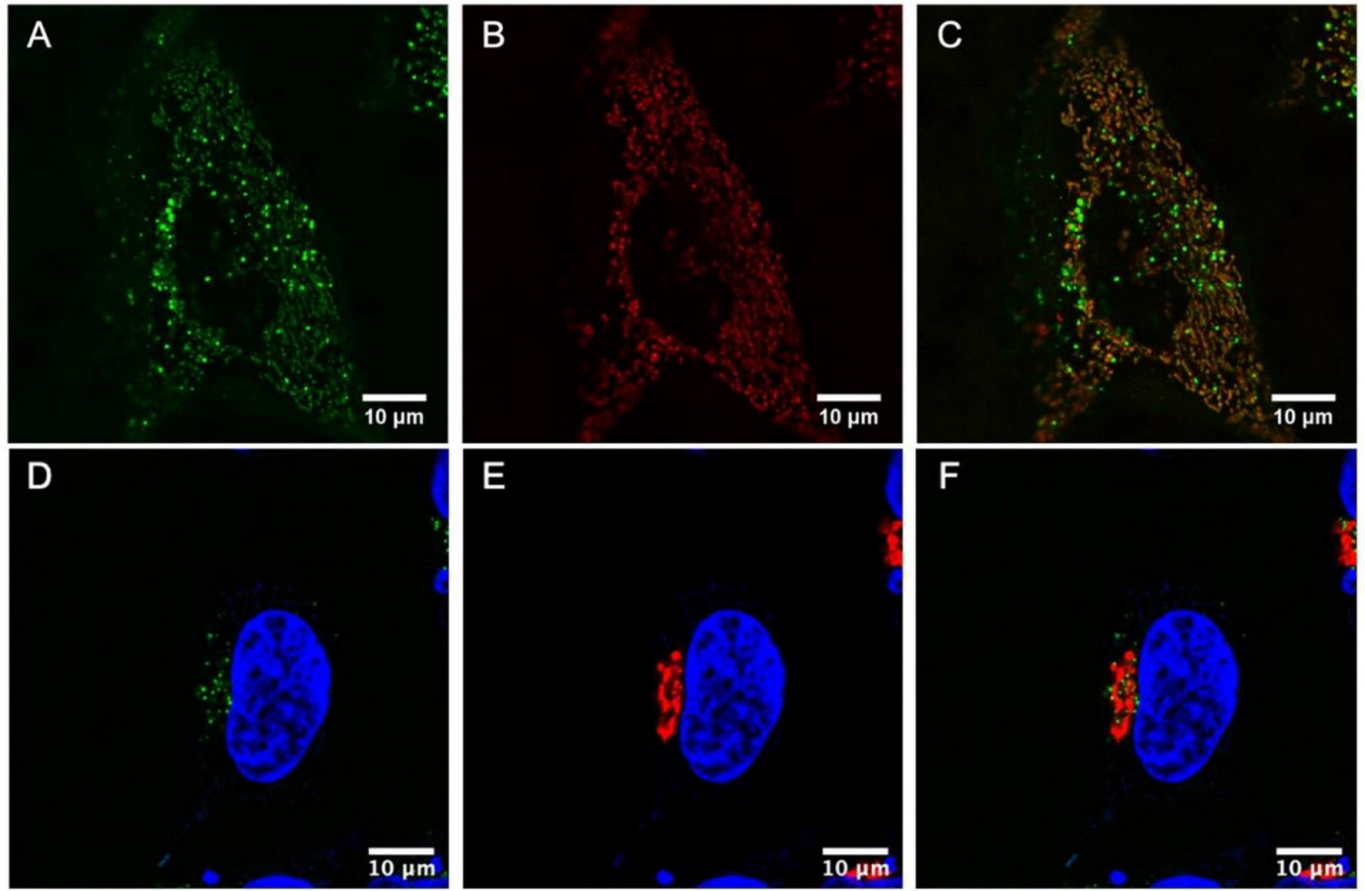
Colocalization studies in live cells. HeLa cells were stained with Ru(dpp)_2_(16-ATAP)]^2+^ (2.5 μM, 24 h) **(A)**, then MitoTracker Deep Red (80 nM) was added to the cells **(B)**, and their colocalization was confirmed **(C)**. HeLa cells were stained with [Ru(dpp)_2_(11-ATAP)]^2+^ (2.5 μM, 24 h) and DAPI (550 nM) **(D)**. Co-staining with CellLight Golgi-RFP (30 μM in 2 mL) **(E)** and overlay of the two channels revealed their strong colocalization **(F)**.

Colocalization studies were then carried out to better understand the very discrete localization of the the probes within living HeLa cells. Figures 5A–C shows a live HeLa cell stained with Ru(dpp)_2_(16-ATAP)]^2+^ in green (A) and co- stained with MitoTracker Deep Red (80 nM) in red (B). Figure 5C overlays the two probes and confirms their very strong colocalization at the mitochondria, reflected in the combined orange color (C). Thus, Ru(dpp)_2_(16-ATAP)]^2+^ is very selectively targeting and localizing at the mitochondria.

Figures 5D–F shows a live HeLa cell stained with DAPI, a nuclear stain for reference (blue) where [Ru(dpp)_2_(11-ATAP)]^2+^ in green (D), co-stained with Golgi-RFP in red (E), and their co-localization (F). The strong colocalization of [Ru(dpp)_2_(11-ATAP)]^2+^ at the mitochondria and Golgi apparatus is remarkable, the origin of the effect is not completely clear as whereas the carbon chain length is different the effect may come from the size of the aggregates of each species to yield very different yet selective targeting can be achieved.

Co-localization studies were also carried out in CHO cells. The punctate staining patterns of [Ru(dpp)_2_(16-ATAP)]^2+^ in CHO cells suggested localization in ER or possibly lipid droplets, given the lipophilicity of the complexes. When incubated with [Ru(dpp)_2_(16-ATAP)]^2+^ Nile Red, a commercial stain for lipid droplets and ER Tracker Blue, some but not exclusive co-localization was observed (ESI Figure S12). This suggests that Ru(dpp)_2_(16-ATAP)]^2+^ has some but not exclusive affinity for lipid rich regions in CHO cells. However, when incubated with [Ru(dpp)_2_(11-ATAP)]^2+^ and a series of commercial probes (Lysotracker Green, ER-Tracker Blue and Golgi-RFP), no co-localization was observed, indicating that [Ru(dpp)_2_(11-ATAP)]^2+^ in CHO cells shows no particular affinity for any specific organelle. Its appearance as punctate spots is likely to be due to retention in endosomes as a result of endocytosis, eventually localizing in the cytoplasm. A Pearson's coefficient for LysoTracker was obtained and found to be 0.28, indicating no co-localization in the lysosomes. The different localization patterns between HeLa cells and CHO cells is interesting, and may be explained by the difference in organelle membrane potential between cancerous and non-cancerous cell types, in particular, the mitochondrial membrane potential (Liu et al., 2019). That the two complexes which differ only in length of lipid tail distributes so differently in both in single and different cell types is also notable, and given they are vesicular aggregates may be attributed to differences in charge or aggregate size rather than the thioacetate tails appended on the complexes.

The parent amino complex was not soluble enough to study in aqueous media as the aggregates were but did permeate and distribute throughout the cytoplasm non-specifically and was nuclear excluding, when dissolved in 0.5% DMSO solution used in the staining procedure.

## Conclusions

In summary, the synthesis and characterization, both photophysical and electrochemical, of a new family of Ruthenium complexes, [Ru(dpp)_2_(x-ATAP)]^2+^, where x-ATAP is 5-amino-1,10-phenanthroline with pendant alkyl-acetylthio chains of varying length; and *x* = 6, 8, 11, and 16 has been reported. Soluble in organic media, the complexes form homogenous solutions with long-lived oxygen dependent emission centered around 600 nm. In aqueous media, the complexes form nanoaggregates confirmed by dynamic light scattering studies with diameter, that depends on chain length and media, of between 140 and 180 nm, indicating that they are bicellar or multivesiclar. The complex aggregates were found to readily permeate the cell membrane of HeLa and CHO cells in a temperature dependent manner, attributed to endocytosis. Notably, specific and selective organelle localization was found to be alkyl chain length and cell line dependent. In HeLa cells, highly discrete localization of [Ru(dpp)_2_(11-ATAP)]^2+^ to the Golgi apparatus was observed whereas the [Ru(dpp)_2_(16-ATAP)]^2+^ accumulates in the mitochondria. Conversely in CHO cells [Ru(dpp)_2_(16-ATAP)]^2+^ was found to have a strong affinity for lipid rich lipid droplets while [Ru(dpp)_2_(11-ATAP)]^2+^ distributes as punctate features throughout the cytoplasm, thought to be endosomes. Overall, the study demonstrates that driving such metal complexes into self-aggregated vesicles provides a pathway for cell permeation and that it may also provide a route for targeted localization without the need for expensive biomolecular conjugation.

## Data Availability Statement

The raw data supporting the conclusions of this article will be made available by the authors, without undue reservation.

## Author Contributions

TK conceived the project. TK, SF, and AB designed the experiments. SF performed synthesis and characterization and spectroscopic and electrochemical studies. KG performed Concentration dependent emission studies and Light scattering studies. AB and SF preformed cell based studies. The manuscript was written by SF and TK with input from KG and AB and approved by all authors before submission. All authors contributed to the article and approved the submitted version.

## Conflict of Interest

The authors declare that the research was conducted in the absence of any commercial or financial relationships that could be construed as a potential conflict of interest.
