# Supplementary material for: Photophysics and Cell Uptake of Self-Assembled Ru(II)Polypyridyl Vesicles
Source: Front Chem. 2020 Jul 30;8:638. doi: 10.3389/fchem.2020.00638 (PMC7406788; doi:10.3389/fchem.2020.00638)
Supplement: Supplementary file 1 [file Table_1.DOCX]

Supplementary Material

Scheme 1: Preparation of [Ru(dpp)2(x-ATAP)](PF6)2 complexes

# Synthesis

*Synthesis of* *4-(4,6-Dimethoxy-1,3,5-triazin-2-yl)-4-methyl-morpholinium (DMTMM)*

2-Chloro-4,6-dimethoxy-1,3,5-triazine (0.0483 g, 2.5 x 10-4 mol) was dissolved in 1ml THF. N-methylmorpholine (0.0253 g, 2.5x 10-4 mol) was added to this stirring solution. This solution was stirred for 30 minutes at room temperature. The white precipitate was collected by vacuum filtration and washed with THF before collecting the dried product. Structure and purity was analysed by 1­HNMR analysis. 1H NMR in deuterated DMSO. 4.35 (d, 2H), 4.1 (s, 6H), 3.98 (d, 2H), 3.9 (t, 2H), 3.77 (t, 2H), 3.47 (s, 3H).

*Synthesis of 6-(acetylthio)-hexanoic acid*

800 mg potassium thioacetate was added to a stirred solution of 6-bromohexanoic acid (0.468 g, 2.4 mmol) in 16 ml anhydrous DMF at 0o C. The solution was stirred at room temperature for 30 minutes, diluted with 40 ml CH2Cl2 and washed three times with water. The organic solution was dried with MgSO4 and the solvent was removed by rotary evaporation. A yellow oil was collected. 0.1414 g (31 % yield) of 6-(acetylthio)-hexanoic acid was recovered. Structure and purity was confirmed by 1­HNMR analysis. 1H NMR in deuterated CDCl3. 2.86 (t, 2H), 2.35 (t, 2H), 2.32 (s, 3H), 1.62 (m, 4H), 1.42 (m, 2H).

*Synthesis of 8-(acetylthio)-octanoic acid*

400 mg potassium thioacetate was added to a stirred solution of 8-bromooctanoic acid (0.267 g, 1.2 mmol) in 8 ml anhydrous DMF at 0o C. The solution was stirred at room temperature for 30 minutes, diluted with 20 ml CH2Cl2 and washed three times with water. The organic solution was dried with MgSO4 and the solvent was removed by rotary evaporation. A yellow oil that solidified on standing was collected. 0.1355 g (51.7 % yield) of 8-(acetylthio)-octanoic acid was recovered. Structure and purity was confirmed by 1­HNMR analysis. 1H NMR in deuterated CDCl3. 2.86 (t, 2H), 2.35 (t, 2H), 2.32 (s, 3H), 1.63 (m, 2H), 1.56 (m, 2H), 1.33 (m, 6H)

*Synthesis of 11-(acetylthio)-undecanoic acid*

11-Mercaptoundecanoic acid (0.1899 g, 0.87 mmol) was dissolved in 3 ml of dichloromethane and 3 ml of acetic acid. 0.5 g zinc powder was then added to the stirred solution. The solution was then stirred for 20 minutes. After this time the reaction solution was cooled to 0o C and 1.2 ml (17 mmol) of acetyl chloride was added. When production of hydrogen gas ceased, after about 20 minutes, the reaction solution was allowed to reach room temperature. After 10 minutes the zinc powder was removed by filtering the reaction solution through celite. The reaction solution was then washed with HCl (0.1 M, 25 ml) and mixed with ice twice, each time collecting the organic phase. The solvent was then removed and was purified by flash chromatography through silica. This was repeated twice, the first time using toluene/ethyl acetate 100:1 as the mobile phase and the second time using toluene/ethyl acetate 10:1. 1­HNMR data showed no difference in product purity after purification with flash chromatography so this step was left out in subsequent reactions. 0.1648 g (72 % yield) of 11-(acetylthio)-undecanoic acid was recovered. 1H NMR in deuterated CDCl3. 2.86 (t, 2H), 2.36 (t, 2H), 2.33 (s, 3H), 1.63 (m, 2H), 1.56 (m, 2H), 1.27 (m, 12H)

*Synthesis of 16-(acetylthio)-hexadecanoic acid*

16-mercaptohexadecanoic acid (0.5 g, 1.74 mmol) was dissolved in 6 ml of dichloromethane and 6 ml of acetic acid. 1 g zinc powder was then added to the stirred solution. The solution was then stirred for 20 minutes. After this time the reaction solution was cooled to 0o C and 2.4 ml (17 mmol) of acetyl chloride was added. When production of hydrogen gas ceased after about 20 minutes the reaction solution was allowed to reach room temperature. After 10 minutes the zinc powder was removed by filtering the reaction solution through a celite column. The reaction solution was then washed with HCl (0.1 M, 50 ml) mixed with ice twice, each time collecting the organic phase. The organic phase was rotary evaporated to dryness and collected. 0.5355 g (93.1 % yield) of 16-(acetylthio)-hexadecanoic acid was recovered. Structure and purity was confirmed by 1­HNMR analysis. 1H NMR in deuterated CDCl3. 2.86 (t, 2H), 2.35 (t, 2H), 2.32 (s, 3H), 1.63 (m, 2H), 1.56 (m, 2H), 1.25 (m, 22H)

*Synthesis of [Ru(dpp)2]Cl2*

0.1181 g (4.52 x 10-4 mol) RuCl3.3(H2O) was dissolved in 15 ml of DMF and stirred. 0.2 g of LiCl was added to the stirring solution. The solution was then brought to reflux under a nitrogen atmosphere. 0.3 g (9.04 x 10-4 mol) of bathophenanthroline was then added slowly to the refluxing solution over 20 minutes. The solution was refluxed under a nitrogen atmosphere for 8 hours. After this time the reaction was cooled and added to 250 ml of acetone and kept in a freezer for 16 hours. The precipitate that had formed was collected by vacuum filtration. The precipitate was then washed with water, to remove any [Ru(dpp)3]Cl2, until the water ran clear. The precipitate was dried with a small amount of diethyl ether. Structure and purity was confirmed by HPLC and 1­HNMR analysis. 0.1020 g (27 % yield) of [Ru(dpp)2]Cl2 was recovered. 1H NMR in deuterated DMSO. 10.44 (d, 2H), 8.26 (dd, 4H), 8.06 (dd, 4H), 7.85 (d, 4H), 7.74, (t, 4H), 7.72 (t, 2H), 7.56 (m, 10H), 7.42 (d, 2H)

*Synthesis of 5-amino-1,10-phenanthroline*

1.5 g (6.6 x 10-3 mol) of 5-nitro-1,10 phenanthroline was dissolved in 30 ml of EtOH. 0.3 g of 5 % Pd/C catalyst was added to the solution and the solution was heated to 70o C under an N2 atmosphere. 1.55 g of hydrazine monohyrdrate was added dropwise to the solution over 30 minutes. The solution was then stirred for 5 hours at 70 oC. The solution was then cooled to room temperature and filtered over celite to remove the catalyst which was washed with EtOH to remove any residual product. The filtrate was rotary evaporated until the first yellow crystals formed. The solution was then diluted with just enough EtOH to redissolve the crystals. The solution was filtered to remove the grey impurity and left over night to crystallize. The precipitate that formed was filtered off and washed with water and a small amount of diethyl ether. 0.7071 g (55 % yield) of 5-amino-1,10-phenanthroline was collected. Purity was confirmed with 1­HNMR analysis. 1H NMR in deuterated DMSO. 9.04 (quintet, 1H), 8.66 (quintet, 2H), 8.03 (dd, 1H), 7.73 (quintet, 1H), 7.75 (quintet, 1H), 6.86 (s, 1H), 6.1 (s, 2H)

*Synthesis of 5-Amido-1,10-phenanthroline-(6-acetylthio-hexanyl)*

220 mg (1.132 mmol) of NH2phen and 195 mg (1.029 mmol) of 6-(acetylthio)-hexanoic acid were dissolved in 15ml anhydrous DMF and stirred for 30 minutes. 2 equivalents (0.5695 g, 2.058 mmol) DMTMM was added to the reaction which was then stirred for 18 hrs. When the reaction was completed the solution volume was reduced to about 5 ml by rotary evaporation and then added to 100 ml H2O. The resulting precipitate was collected by vacuum filtration and washed with plenty of water and diethyl ether. The crude solid was sonicated in 50 ml acetone and the undissolved material was removed by vacuum filtration. This was carried out multiple times on the precipitate until the product was found to be sufficiently pure as confirmed by 1­HNMR analysis. 55.4 mg was recovered (13.3 % yield). Structure and purity was confirmed by 1­HNMR analysis. The 1­HNMR numbering scheme is shown in Fig 3.3.3.1. 1H NMR in deuterated DMSO. 10.12 (1H5, s, NH), 9.13 (1H8, dd, C-H), 9.03 (1H1, dd, C-H), 8.60 (1H6, dd, C-H), 8.44 (1H3, dd, C-H), 8.17 (1H4, s, C-H), 7.83 (1H7, dd, C-H), 7.74 (1H2, dd, C-H), 2.87 (2H17-18, t, CH2), ~2.5 obscured by solvent peak (2H9-10, t, CH2), 2.3 (3H19-21, s, CH3), 1.69 (2H15-16, quintet, CH2), 1.58 (2H11-12, quintet, CH2), 1.44 (2H13-14, quintet, CH2).

*Synthesis of 5-Amido-1,10-phenanthroline-(8-acetylthio-octanyl)*

220 mg (1.132 mmol) of NH2phen and 223.9 mg (1.029 mmol) of 8-(acetylthio)-octanoic acid were dissolved in 15 ml anhydrous DMF and stirred for 30 minutes. 2 equivalents (0.5695 g, 2.058 mmol) DMTMM was added to the reaction which was then stirred for 18 hrs. When the reaction was completed the solution was added to 100 ml H2O and the resulting precipitate was collected by vacuum filtration and washed with plenty of water and diethyl ether. The crude solid was sonicated in 50 ml acetone and the undissolved material was removed by vacuum filtration. This was carried out multiple times on the precipitate until the product was found to be sufficiently pure as confirmed by 1­HNMR analysis. 0.1562 g was recovered (34.9 % yield). GC-MS: 418.2 (M+, +23). Structure and purity was confirmed by 1­HNMR analysis. 1H NMR in deuterated DMSO. 10.12 (1H, s, NH), 9.12 (1H, dd, C-H), 9.03 (1H, dd, C-H), 8.60 (1H, dd, C-H), 8.44 (1H, dd, C-H), 8.17 (1H, s, C-H), 7.83 (1H, dd, C-H), 7.74 (1H, dd, C-H), 2.84 (2H, t, CH2), ~2.5 obscured by solvent peak (2H, t, CH2), 2.31 (3H, s, CH3), 1.68 (2H, quintet, CH2), 1.52 (2H, quintet, CH2), 1.4-1.2 (6H, m, three CH2).

*Synthesis of 5-Amido-1,10-phenanthroline-(11-acetylthio-undecanyl)*

220 mg (1.132 mmol) of NH2phen and 266.7 mg (1.029 mmol) of 11-(acetylthio)-undecanoic acid were dissolved in 15 ml anhydrous DMF and stirred for 30 minutes. 2 equivalents (0.5695 g, 2.058 mmol) DMTMM was added to the reaction which was then stirred for 18 hrs. When the reaction was completed the solution was added to 100 ml H2O and the resulting precipitate was collected by vacuum filtration and washed with plenty of water and diethyl ether. The crude solid was sonicated in 50 ml acetone and the undissolved material was removed by vacuum filtration. This was carried out multiple times on the precipitate until the product was found to be sufficiently pure as confirmed by 1­HNMR analysis. 0.1079 g was recovered (21.8 % yield). Structure and purity was confirmed by 1­HNMR analysis. 1H NMR in deuterated DMSO. 10.12 (1H, s, NH), 9.12 (1H, dd, C-H), 9.03 (1H, dd, C-H), 8.60 (1H, dd, C-H), 8.44 (1H, dd, C-H), 8.17 (1H, s, C-H), 7.83 (1H, dd, C-H), 7.74 (1H, dd, C-H), 2.80 (2H, t, CH2), ~2.5 obscured by solvent peak (2H, t, CH2), 2.3 (3H, s, CH3), 1.69 (2H, quintet, CH2), 1.48 (2H, quintet, CH2), 1.4-1.2 (12H, m, six CH2).

*Synthesis of 5-Amido-1,10-phenanthroline-(16-acetylthio-hexadecanyl)*

220 mg (1.132 mmol) of NH2phen and 340 mg (1.029 mmol) of 16-(acetylthio)-hexadecanoic acid were dissolved in 15 ml anhydrous DMF and stirred for 30 minutes. 2 equivalents (0.5695 g, 2.058 mmol) DMTMM was added to the reaction which was then stirred for 18 hrs. When the reaction was completed the solution was added to 100 ml H2O and the resulting precipitate was collected by vacuum filtration and washed with plenty of water and diethyl ether. The crude solid was sonicated in 50 ml acetone and the undissolved material was removed by vacuum filtration. This was carried out multiple times on the precipitate until the product was found to be sufficiently pure as confirmed by 1­HNMR analysis. 0.1213 g was recovered (21.1 % yield). Structure and purity was confirmed by 1­HNMR analysis. 1H NMR in deuterated DMSO. 10.12 (1H, s, NH), 9.12 (1H, dd, C-H), 9.03 (1H, dd, C-H), 8.60 (1H, dd, C-H), 8.44 (1H, dd, C-H), 8.17 (1H, s, C-H), 7.83 (1H, dd, C-H), 7.74 (1H, dd, C-H), 2.80 (2H, t, CH2), ~2.5 obscured by solvent peak (2H, t, CH2), 2.3 (3H, s, CH3), 1.69 (2H, quintet, CH2), 1.47 (2H, quintet, CH2), 1.4-1.2 (22H, m, eleven CH2).

*Synthesis of [Ru(dpp)2(AmidoPhen-6-(acetylthio)-hexanyl)](PF6)2*

0.0554 g (1.5065x10-4 mol) Amidophenanthroline-6-(acetylthio)-hexanyl and 0.1315 g [Ru(dpp)2]Cl2 were dissolved in 50 ml 80 : 20 EtOH : H20. This solution was refluxed for 16 hrs and then the reaction mixture was rotary evaporated down to about 15 ml. Ammonium hexafluorophosphate was used to precipitate the product as a PF6 salt. The orange product was collected by vacuum filtration and washed with water and diethyl ether. 0.1485 g was recovered (69 % yield). Structure and purity was confirmed by 1­HNMR analysis. GC-MS: 1278 (M+ -145). 1H NMR in deuterated DMSO. 10.46 (1H, s, N-H), 8.92 (1H, d, C-H), 8.79 (1H, d, C-H), 8.67 (1H, s, C-H), 8.35 (1H, d, C-H), 8.32 (1H, d, C-H), 8.27 (5H, m, C-H), 8.20 (2H, m, C-H), 8.14 (1H, t, C-H), 7.90 (1H, m, C-H), 7.82 (3H, m, C-H), 7.8 (2H, m, C-H), 7.7-7.5 (20H, m, C-H), 2.86 (2H, t, CH2), 2.60 (2H, t, CH2), 2.30 (3H, s, CH3), 1.67 (2H, quintet, CH2), 1.59 (2H, quintet, CH2), 1.44 (2H, quintet, CH2). Elemental analysis: C 57.38 % (56.96 %), H 3.75 % (3.58 %), N 6.89 % (6.68 %). Calculated values are in brackets.

*Synthesis of [Ru(dpp)2(AmidoPhen-8-(acetylthio)-octanyl)](PF6)2*

75 mg (1.898x10-4 mol) Amidophenanthroline-8-(acetylthio)-octanyl and 0.1657 g [Ru(dpp)2]Cl2 were dissolved in 50 ml 80 : 20 EtOH : H20. This solution was refluxed for 16 hrs and then the reaction mixture rotary evaporated down to about 15 ml. Ammonium hexafluorphosphate was used to precipitate the product as a PF6 salt. The orange product was collected by vacuum filtration and washed with water and diethyl ether. 0.242 g was recovered (87.8 % yield). Structure and purity was confirmed by 1­HNMR analysis. GC-MS: 1305.8 (M+, -145). 1H NMR in deuterated DMSO. 10.46 (1H, s, N-H), 8.91 (1H, d, C-H), 8.78 (1H, d, C-H), 8.66 (1H, s, C-H), 8.35 (1H, d, C-H), 8.32 (1H, d, C-H), 8.28 (5H, m, C-H), 8.20 (2H, m, C-H), 8.14 (1H, t, C-H), 7.90 (1H, m, C-H), 7.82 (3H, m, C-H), 7.8 (2H, m, C-H), 7.7-7.5 (20H, m, C-H), 2.83 (2H, t, CH2), 2.59 (2H, t, CH2), 2.30 (3H, s, CH3), 1.69 (2H, quintet, CH2), 1.51 (2H, quintet, CH2), 1.45-1.2 (6H, m, CH2). Elemental analysis: C 57.93 % (57.62 %), H 3.96 % (3.76 %), N 6.76 % (6.56 %). Calculated values are in brackets.

*Synthesis of [Ru(dpp)2(AmidoPhen-11-(acetylthio)-undecanyl)](PF6)2*

53 mg (1.288x10-4 mol) Amidophenanthroline-11-(acetylthio)-undecanyl and 0.1123 g [Ru(dpp)2]Cl2 were dissolved in 50 ml 80 : 20 EtOH : H20. This solution was refluxed for 16 hrs and then the reaction mixture was rotary evaporated down to about 15 ml. Ammonium hexafluorophosphate was used to precipitate the product as a PF6 salt. The orange product was collected by vacuum filtration and washed with water and diethyl ether. 0.1666 g was recovered (86.7 % yield). Structure and purity was confirmed by 1­HNMR analysis. GC-MS: 1348 (M+, -145). 1H NMR in deuterated DMSO. 10.58 (1H, s, N-H), 8.91 (1H, d, C-H), 8.78 (1H, d, C-H), 8.67 (1H, s, C-H), 8.35 (1H, d, C-H), 8.32 (1H, d, C-H), 8.27 (5H, m, C-H), 8.20 (2H, m, C-H), 8.14 (1H, t, C-H), 7.90 (1H, m, C-H), 7.82 (3H, m, C-H), 7.8 (2H, m, C-H), 7.7-7.5 (20H, m, C-H), 2.79 (2H, t, CH2), 2.63 (2H, t, CH2), 2.29 (3H, s, CH3), 1.70 (2H, quintet, CH2), 1.46 (2H, quintet, CH2), 1.45-1.2 (12H, quintet, CH2). Elemental analysis: C 58.71 % (56.15 %), H 4.25 % (3.89 %), N 6.57 % (6.03 %). Calculated values are in brackets.

*Synthesis of [Ru(dpp)2(AmidoPhen-16-(acetylthio)-hexadecanyl)](PF6)2*

55.1 mg (1.084x10-4 mol) Amidophenanthroline-16-(acetylthio)-hexadecanyl and 94.6 mg [Ru(dpp)2]Cl2 were dissolved in 50 ml 80 : 20 EtOH : H20. This solution was refluxed for 16 hrs and then the reaction mixture was rotary evaporated down to about 15 ml. Ammonium hexafluorophosphate was used to precipitate the product as a PF6 salt. The orange product was collected by vacuum filtration and washed with water and diethyl ether. 0.1475 g was recovered (87 % yield). Structure and purity was confirmed by 1­HNMR analysis. GC-MS: 1418 (M+, -145). 1H NMR in deuterated DMSO. 10.45 (1H, s, N-H), 8.91 (1H, d, C-H), 8.78 (1H, d, C-H), 8.68 (1H, s, C-H), 8.35 (1H, d, C-H), 8.32 (1H, d, C-H), 8.26 (5H, m, C-H), 8.20 (2H, m, C-H), 8.14 (1H, t, C-H), 7.90 (1H, m, C-H), 7.82 (3H, m, C-H), 7.8 (2H, m, C-H), 7.7-7.5 (20H, m, C-H), 2.78 (2H, t, CH2), 2.67 (2H, t, CH2), 2.29 (3H, s, CH3), 1.70 (2H, quintet, CH2), 1.46 (2H, quintet, CH2), 1.44 (22H, quintet, CH2). Elemental analysis: C 57.38 % (56.96 %), H 3.75 % (3.58 %) N 6.89 % (6.68 %). Calculated values are in brackets. Elemental analysis: C 59.92 % (58.96 %), H 4.71 % (4.44 %), N 6.27 % (5.84 %). Calculated values are in brackets.

*Synthesis of [Ru(dpp)2(NH2phen)].(PF6)2*

0.0779 g of (8.9 x 10-5 mol) of [Ru(dpp)2].Cl2 and 0.0182 g (8.9 x 10-5 M) of NH2phen were dissolved in 50 ml of 80 : 20 EtOH : H2O. This solution was refluxed for 16 hrs and then the reaction mixture was rotary evaporated down to dryness. The orange product was collected. 0.086 g was recovered (90 % yield). This dichloride product was converted to a (PF6)2 salt by dissolving [Ru(dpp)2(NH2phen)].(Cl)2 in a saturated solution of ammonium hexafluorophosphate and collecting the resulting precipitate by vacuum filtration. 1H NMR in deuterated DMSO. 1H NMR in deuterated DMSO. 9.01 (d, 1H), 8.35 (m, 3H), 8.26 (s, 4H), 8.19 (m, 3H), 7.79 (m, 5H), 7.47 (d, 1H), 7.66 (m, 21H), 7.14 (s, 1H), 7.05 (s, 2H)

**Table S1:** Solution phase electrochemistry data for RuxD complexes All solution phase electrochemistry was carried out with 1 mM solutions of complex in ACN with 0.1 mM TBATBF4 as the supporting electrolyte and a glassy carbon electrode. All potentials are quoted vs. Ag/AgNO3 electrode.

|  | E1/2­ red ligand | E1/2 ox Ru2+/3+ | ΔEpox Ru2+/3+ |
| --- | --- | --- | --- |
| Ru6D | -1.7 V | +0.92 V | 0.08 V |
| Ru8D | -1.7 V | +0.92 V | 0.085 V |
| Ru11D | -1.67 V | +0.92 V | 0.081 V |
| Ru16D | -1.67 V | +0.92 V | 0.081 V |

# Supplementary Figures and Tables

**Figure S1:** 1H NMR of [Ru(dpp)2(AmidoPhen-6-(acetylthio)-hexanyl)(PF6­)2]2+ in (CD3)2SO.

*
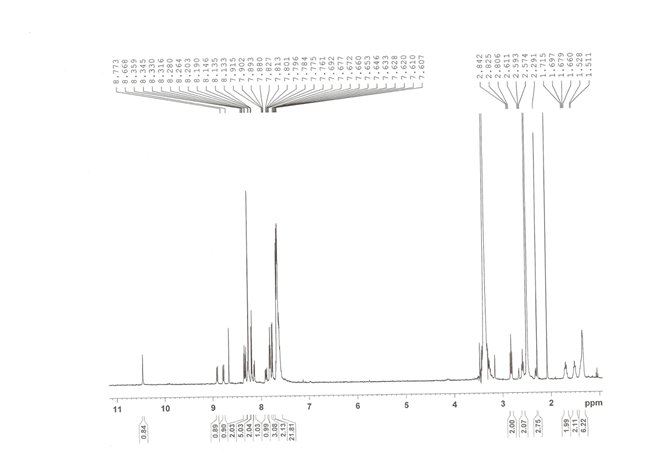
*

**Fig S2:** 1H NMR of [Ru(dpp)2(AmidoPhen-8-(acetylthio)-octanyl)(PF6)2]2+ in (CD3)2SO.

**Figure S3:** 1H NMR of [Ru(dpp)2(AmidoPhen-11-(acetylthio)-undecanyl)(PF6)2]2+ in (CD3)2SO

**Figure S4:** 1H NMR of [Ru(dpp)2(AmidoPhen-16-(acetylthio)-hexadecanyl)(PF6)2]2+ in (CD3)2SO.

10% w/w in KBr discs. An excitation wavelength of 458 nm was used.

**Figure S5:** Solution phase resonance Raman spectrocopy of [Ru(dpp)2(x-ATAP)]2+ where x is bottom to top, 6, 8, 11, 16. Samples were prepared as 5e-5 M in ACN and excited at 457.8 nm using an Argon Ion laser. The features are predominantly diphenyl phenanthroline indicating the excited state at this excitation is Ru to DPP transition.

Figure S6: Luminescence decay for [Ru(dpp)2(16-ATAP)]2+ in DPPC liposomes. The DPPC liposomes were suspended in water. The black line and residuals show the fit to biexponential kinetic trace.


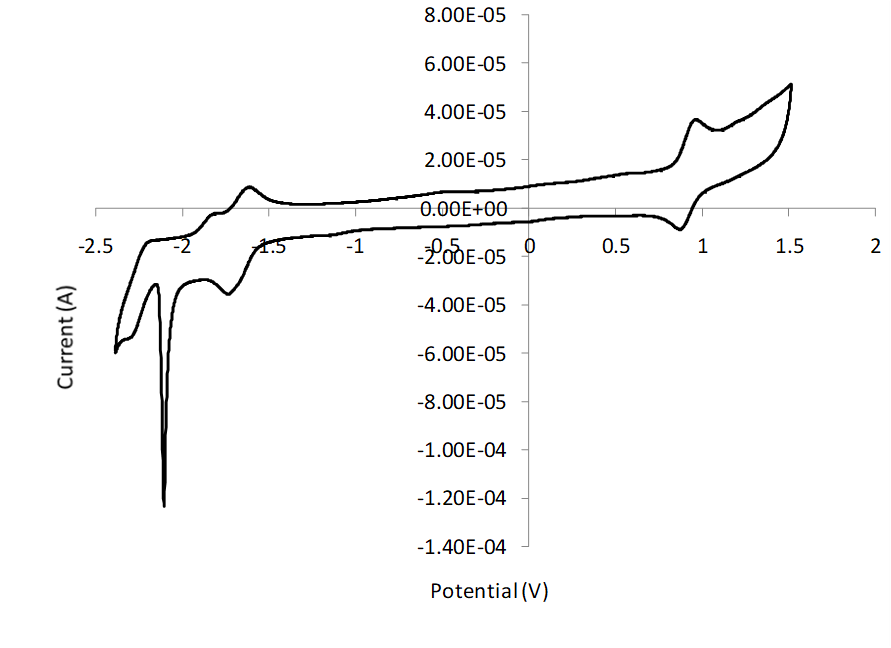


Figure S7: Cyclic Voltammagram of 1mM [Ru(dpp)2(16-ATAP)]2+ in ACN with 0.1 mM TBATBF4 as supporting electrolyte; glassy carbon electrode is the working electrode, Pt auxiliary electrode and potentials are vs. Ag/AgNO3 reference electrode.

**Figure S8:** Representative absorption spectra of [Ru(dpp)2(11-ATAP)]2+ showing impact of solvent on absorption in ACN, DCM and H2O. Concentration of [Ru(dpp)2(11-ATAP)]2+ is 5 x 10-6 M in all solvents. The same effects are observed across all of the complexes explored.


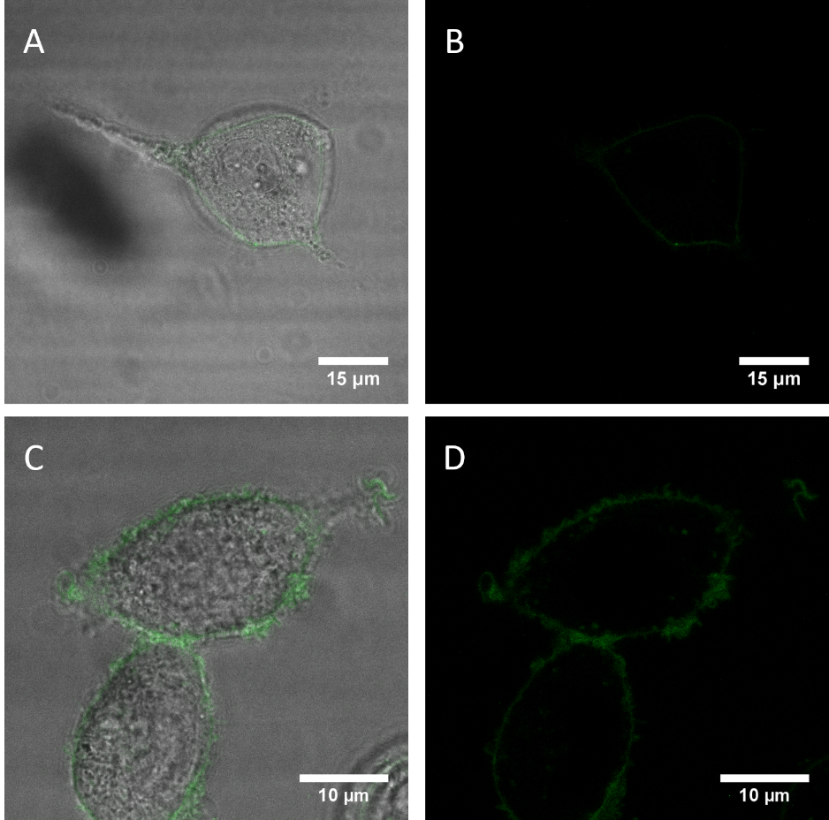


**Figure S9:** Temperature dependence uptake of [Ru(dpp)2(16-ATAP)]2+ (2.5 µM) by live HeLa (A,B) and CHO (C,D) cells at 4°C for 24 h. A,C show overlay of ruthenium emission channel with background fluorescence channel. B,D show emission from ruthenium channel only.


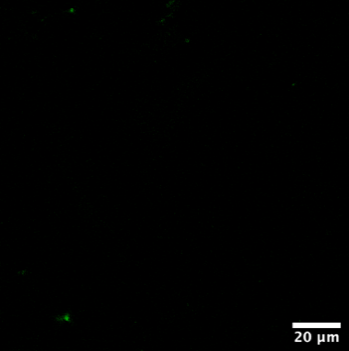

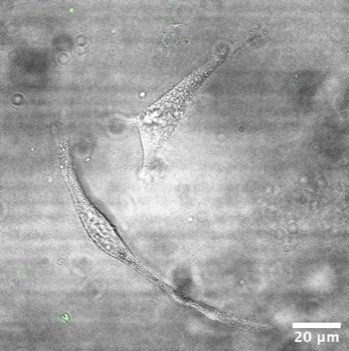

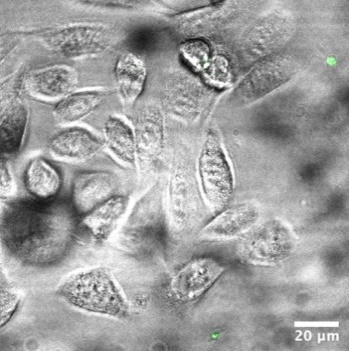

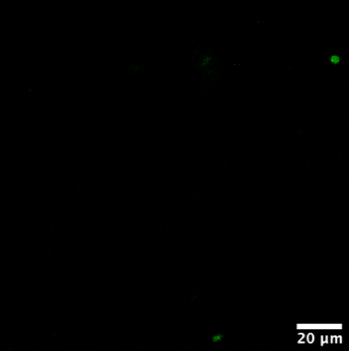


A

D

C

B

**Figure S10:** Temperature dependence uptake of [Ru(dpp)2(11-ATAP)]2+ (2.5 µM) by live HeLa (A,B) and CHO (C,D) cells at 4°C for 24 h. A,C show overlay of ruthenium emission channel with background fluorescence channel. B,D show emission from ruthenium channel only.


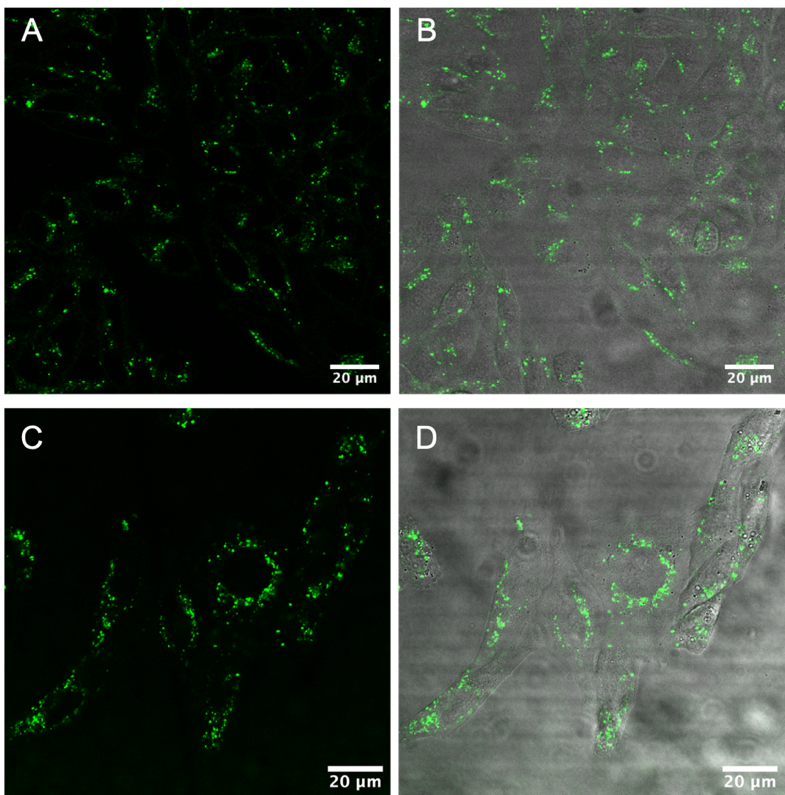


**Figure S11:** Live CHO cells stained with 2.5 μM [Ru(dpp)2(16-ATAP)]2+ (A,B) and [Ru(dpp)2(11-ATAP)]2+ (C,D) for 24 h.


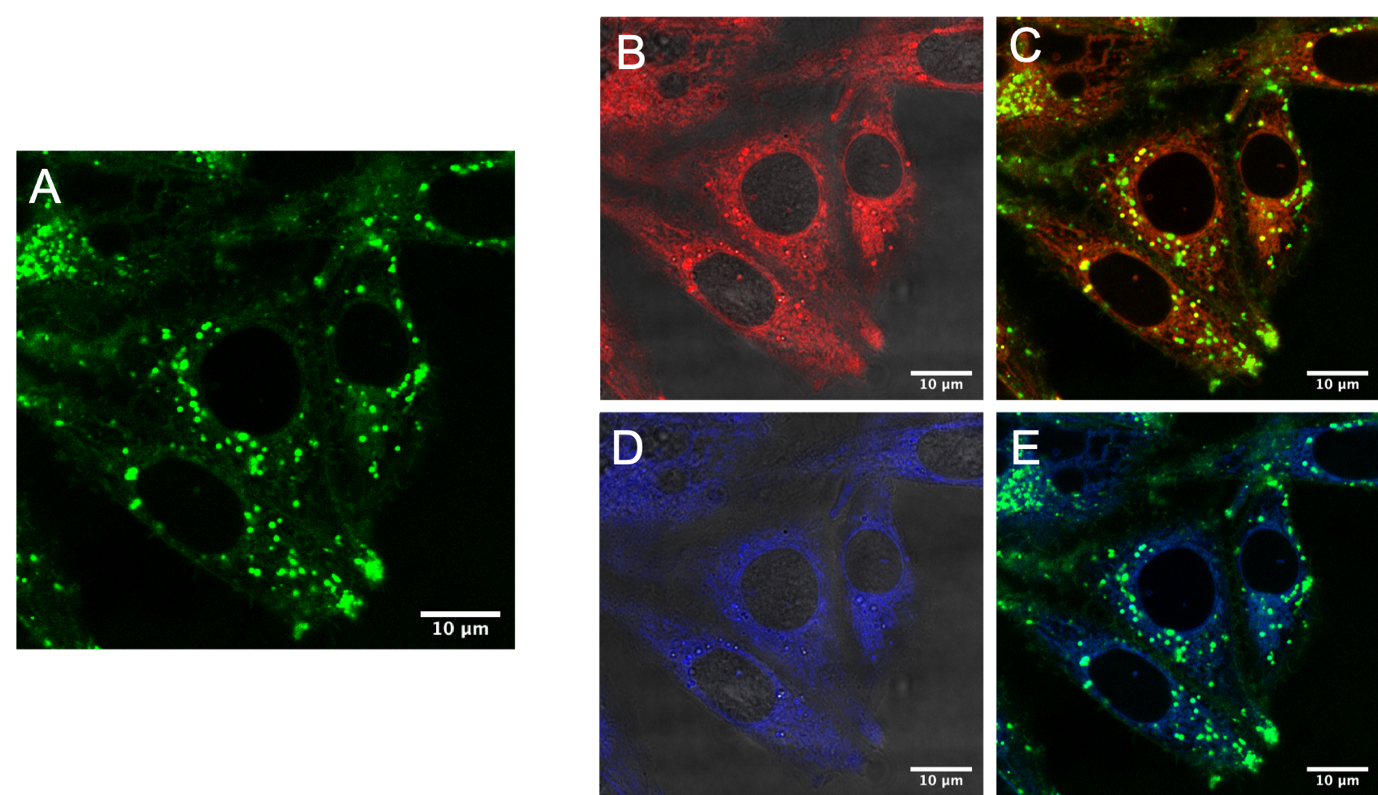


**Figure S12:** Live CHO cells stained with 2.5 μM [Ru(dpp)2(16-ATAP)]2+, 24 h, (A) and co-stained with commercial dyes Nile Red (B) and their overlap (C). Cell were co-stained with ER-Tracker blue (D) and their overlap (E).


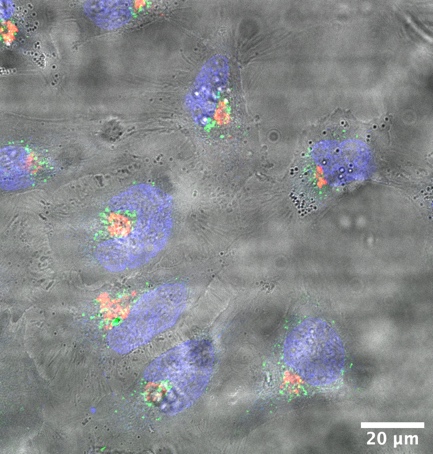

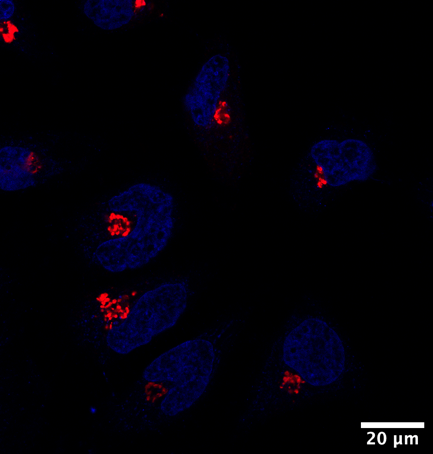

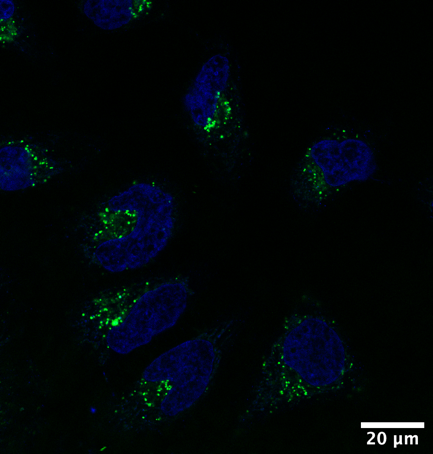

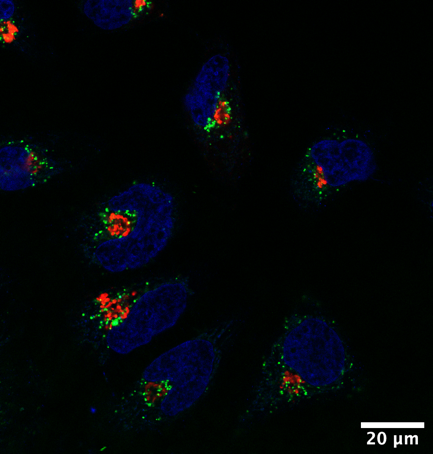


A

B

C

D

**Figure S13:** Live HeLa cells incubated with [Ru(dpp)2(11-ATAP)]2+ (2.5 μM) and Cell Light Golgi-RFP for 24 h. DAPI (500 nM) was added to cells, then removed and washed prior to imaging. Ru11D and DAPI channels (A), Golgi-RFP and DAPI channels (B), overlay of DAPI, Ru11D and Golgi-RFP showing colocalisation in the golgi apparatus (C), and the overlay of all channels with the white light transmission to show cell morphology (D).


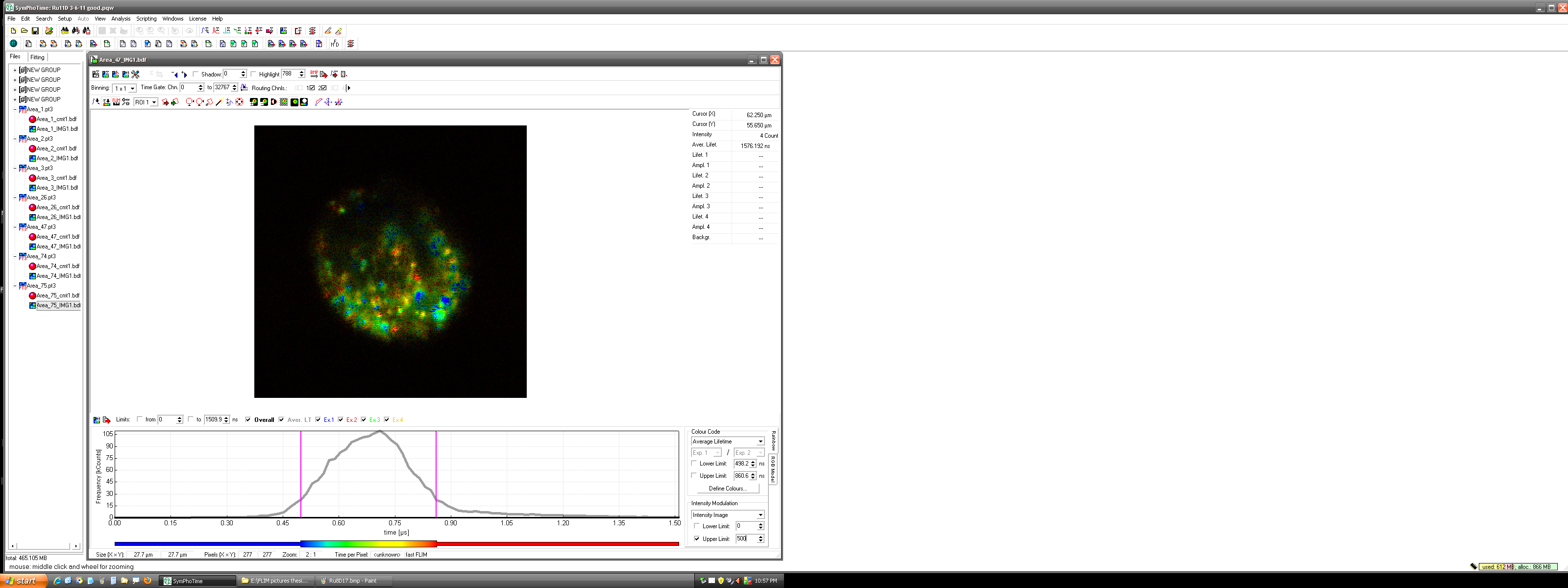


**Figure S14:** Average luminescence lifetime maps of live CHO cell following incubated for 24 hours with [Ru(dpp)2(11-ATAP)]2+. Below lifetime histogram for the image. FLIM analysis was carried out using a Microtime 200 FLIM system. A diode laser provided an excitation wavelength of 440 nm. Samples were mounted and focused using an Olympus IX71 microscope platform and a x60 objective. Data analysis was carried out on Symphotime analysis software.
